# Genetic Susceptibility for Individual Cooperation Preferences: The Role of Monoamine Oxidase A Gene (*MAOA*) in the Voluntary Provision of Public Goods

**DOI:** 10.1371/journal.pone.0020959

**Published:** 2011-06-16

**Authors:** Vanessa Mertins, Andrea B. Schote, Wolfgang Hoffeld, Michele Griessmair, Jobst Meyer

**Affiliations:** 1 Institute for Labour Law and Industrial Relations in the European Community (IAAEG), University of Trier, Trier, Germany; 2 Department of Neurobehavioral Genetics, Institute of Psychobiology, University of Trier, Trier, Germany; 3 Institute for Labour Law and Industrial Relations in the European Community (IAAEG), University of Trier, Trier, Germany; 4 Institute for Labour Law and Industrial Relations in the European Community (IAAEG), University of Trier, Trier, Germany; 5 Department of Neurobehavioral Genetics, Institute of Psychobiology, University of Trier, Trier, Germany; University of Utah, United States of America

## Abstract

In the context of social dilemmas, previous research has shown that human cooperation is mainly based on the social norm of conditional cooperation. While in most cases individuals behave according to such a norm, deviant behavior is no exception. Recent research further suggests that heterogeneity in social behavior might be associated with varying genetic predispositions. In this study, we investigated the relationship between individuals' behavior in a public goods experiment and the promoter-region functional repeat polymorphism in the monoamine oxidase A gene (*MAOA*). In a dynamic setting of increasing information about others' contributions, we analyzed differences in two main components of conditional cooperation, namely the players' own contribution and their beliefs regarding the contribution of other players. We showed that there is a significant association between individuals' behavior in a repeated public goods game and *MAOA*. Our results suggest that male carriers of the low activity alleles cooperate significantly less than those carrying the high activity alleles given a situation where subjects had to rely on their innate beliefs about others' contributions. With increasing information about the others' cooperativeness, the genetic effect diminishes. Furthermore, significant opposing effects for female subjects carrying two low activity alleles were observed.

## Introduction

Antisocial behavior can be conceptualized as a failure to conform to social norms. Social norms are standards of behavior that are based on widely shared beliefs on how individuals ought to behave in particular situations [1]. In the context of social dilemmas [2], human cooperation is mainly based on a social norm of conditional cooperation: cooperate if the others cooperate and defect otherwise. Conditional cooperators thus follow the famous tit-for-tat strategy [3], which underlies many evolutionary models [4]. Individuals that violate cooperative norms choose to defect rather than cooperate, a choice that is always harmful to others.

By now, there is little scientific doubt that both nature and nurture contribute to observed variation in antisocial behavior [5]–[7]. Arguably, the clearest link between this kind of behavioral pattern and allelic variation exists for the monoamine oxidase A gene (*MAOA*) [8], [9]. The gene encodes MAOA, which is a key enzyme responsible for the degradation of neurotransmitters such as serotonin, dopamine and norepinephrin in the brain [10]. Various association studies [11]–[16] confirm that the low enzyme expressing alleles (*MAOA-L*) predict - at least for males - antisocial behavior. We expect that subjects with less transcriptionally-efficient alleles will fail to conform to the social norm of conditional cooperation.

In this work, we refer to voluntary contributions to the provision of public goods as the main variable of interest. We investigated by means of an economic experiment to what extent observed behavior and beliefs about others' behavior are a product of biological as well as environmental factors. Public goods settings are of considerable concern in various fields such as economics [17], biology [18] and psychology [2]. This research has established four basic facts: a) people contribute sizeable shares of their endowments even in situations in which it is a rational strategy to contribute nothing, b) relatively high initial levels of contribution tend to diminish over time, converging towards zero contribution [19], c) multiple behavioral types exist that point to large preference heterogeneity [17], [20], and d) people behave mostly as “conditional cooperators”, i.e., their contributions to the public good depend directly on how group members are believed to behave [1], [21], [22].

Economic experiments may be considered as “atomic measures of economic traits” [23]. In this sense, they are especially suitable to measure individuals' preferences under different environmental conditions, therewith providing the means to assess gene-environment interactions. We used a standard repeated public goods experiment to elicit people's preferences towards cooperation. With respect to genotypes, we followed the targeted association study approach by focusing on a candidate gene, which has previously been linked to related behavioral patterns. Several key empirical findings have motivated our decision to study the association between decisions in public goods experiments and *MAOA*. The catabolic activity of the encoded enzyme has made *MAOA* a very attractive candidate in the study of neurological diseases as well as psychiatric and behavioral traits [24]. The gene encoding the MAOA protein is located on chromosome Xp11.23-11.4 [25] and harbors a genetic length polymorphic repeat (LPR) in its promoter region [26]. The *MAOA-LPR* consists of a 30 bp repeated sequence and is present in 2, 3, 3.5, 4 or 5 copies. Alleles with 3.5 and 4 copies are transcribed more efficiently compared to those with 2, 3, or 5 copies. In most populations, the 3 and 4 repeat alleles are the most common, whereas the 2, 3.5, and 5 copy alleles are rare [16], [26].

Association studies have shown that carriers of the low activity *MAOA* (*MAOA-L*) have a higher vulnerability to develop psychiatric disorders such as antisocial personality disorder, conduct disorder [12], antisocial alcoholism [13]–[15] and panic disorder [16]. Several brain imaging studies were able to link the *MAOA-LPR* to brain function during cognition, emotional arousal and personality tests. In particular, *MAOA-L* predicted hyperresponsiveness of the amygdala, a brain area involved in emotion-processing that may contribute to increased depression and anxiety [27]. Cohort studies in human and non-human primates [26], [28] showed that *MAOA-L* was associated with aggression and antisocial behavior only in combination with childhood maltreatment, highlighting the significance of gene-environment interaction in psychiatry [9], [29]. Recent literature also suggests that individuals with *MAOA-L* are more likely to react with aggression to challenges [30]. The clearest genetic evidence that *MAOA* regulates human behavior has been described by Brunner and colleagues in a Dutch family with manifestation of a complex behavioral syndrome including borderline mental retardation and impulsive aggression. In the affected males, a nonsense mutation resulted in a *MAOA* deficiency and in increased aggressive behavior [8]. It was shown that *MAOA* deficient mice were more aggressive but also showed more efficient emotional learning [31]. More recently, an association between *MAOA* and political behavior was found: individuals with *MAOA-H* were more likely to vote [32]. Furthermore, *MAOA* was investigated in neuroeconomic studies showing that high activity *MAOA* was associated with attitude towards longshot risks [33] and *MAOA-L* with aggression only after provocation in a power-to-take game [34]. These previous observations motivated us to study *MAOA* with respect to social decision-making and cooperation.

Imperfect conditional cooperation [1], [17] is the prevailing social norm in public good settings, i.e., most individuals contribute a little less than what they believe others would do. Due to the finding that beliefs are adapted according to others' contributions observed in the past (and past beliefs) [35], the environmental variation in our setting was implemented by by using a repeated experiment over ten rounds. After each round, subjects were informed about other group members' average contribution. In this way, step by step, subjects received increasing information in the form of feedback about others' contributions. As previously shown [21], an individual's belief in a given period is the weighted average of the belief about others' contributions in the previous period and the observed contributions in the previous period. Thus, the first period of our game was different from all the remaining ones. In the first period, individuals were not yet able to observe others' actual contributions: they had to rely on their innate or “home-grown” beliefs about others' contributions. We expected that if *MAOA* had an effect on individuals' contributions and beliefs, we should observe the effect in the first round of the game. In subsequent periods, adaptive belief learning should take place: subjects update their beliefs based on the belief and the observed others' contributions from the previous round. When the social environment provides additional feedback, individuals can adjust their behavior and expectations accordingly. Thus, the genetic influence is expected to be dominated by the social environment in later rounds.

Recent findings by McDermott et al. [34] pointed out for the first time the importance of the interaction between social environment and genetic predisposition in the context of an economic experiment. The authors show that the *MAOA* gene does not directly code for aggressive behavior, but affects behavior as a consequence of a direct stimulus from the environment. Without denying the particular importance of strong stimuli for genetic differences, we tested the complimentary hypothesis: genes may play a prominent role if there is no information on how one should behave. Our experimental setting allows to correlate *MAOA* and individuals' own willingness and expected willingness of others to cooperate, including the investigation of environmental effects, i.e. increasing feedback about the cooperativeness of others.

## Methods

The experimental setup was based upon the standard public goods paradigm with the classic voluntary contribution mechanism. The design replicated a well-known experimental design [21]: each subject in a group of four had an endowment of twenty points (with a monetary equivalent of 60 Eurocent). The endowment could be split between private and public investment. All subjects played simultaneously and anonymously. The total sum invested in the public good by all subjects was multiplied by a factor of 1.6 and then divided equally among the players, regardless of the individual contribution. Thus, it was collectively rational to contribute everything, but rational, selfish players contributed nothing to the provision of the public good, regardless of what the others did. This means that individuals who behaved according to their economic incentives did not cooperate but free-ride.

Group composition was randomly rematched every round (strangers design) over 10 periods. At the end of each round, subjects were asked to indicate their belief about the average contributions of others. Afterwards, subjects received information about the average contribution of the other group members. Thus, the setting is characterized by increasing information about others' cooperativeness.

We arranged observations into four stages. The first stage reflects a situation where subjects had no information about others' behavior. Therefore, their behavior should be based on innate attitudes and beliefs. As a consequence, behavior in the first round is different from subsequent periods: it discloses whether individuals hold a rather positive or negative view of the society they live in. In the second round, subjects were able to observe one group contribution from the previous round and so on up to the tenth round where they were able to observe behavior from nine previous rounds. In this way, step by step, more information about others' cooperativeness was provided and contributed to a clearer picture of the social environment. We categorized period 1 as a situation of no information (stage 1), periods 2–4 as a situation of low information (stage 2), periods 5–7 as medium information setting (stage 3) and periods 8–10 as high information environment (stage 4). In summary, environmental variation is given by an increase of additional feedback about others' contributions.

We decided to employ mixed gender sessions due to the fact that individuals are asked to engage in social interaction and the interplay of both genders added some realism. Even more important, it is well known that group gender composition affects individual decision-making [36], [37]. Thus, we expected men in all-male groups to behave differently than men in mixed gender groups. As our intention was to study the latter, we studied mixed gender groups, but focused on the behavior of males.

Both men and women were assigned to two groups: male carriers of 4 repeats were assigned to *MAOA-H*, carriers of 3 repeats to *MAOA-L* (Text S1 for *MAOA* genotyping). As most [34], [38] but not all [16] previous studies assigned heterozygous women to the group of *MAOA-H*, we followed their example: The high activity group consisted of 3/4 and 4/4- genotype, the low activity group of 3/3-genotype. This assignment resulted in 33% *MAOA-L* men (which equals the average proportion found in Western European subject pools) and 19% *MAOA-L* women (5 subjects were excluded from the analysis due to genotyping problems). With the help of an ex post questionnaire, we were able to administer background characteristics such as age, field of study, the number of friends within an experimental session and various personality measures. We found that potential differences in behavior between genotypes cannot be attributed to differences in these background characteristics as they do not differ at a 5%-level between MAOA-H and MAOA-L for male and female subjects (Mann-Whitney tests, two-sided).

We hypothesized that subjects hold individual social norms of behavior and individual beliefs about the behavior of others in every round. However, we expected innate beliefs only to be observable in the first period, whereas adapted beliefs are measurable in all subsequent periods. Furthermore, we hypothesized that, first, *MAOA-L* men contribute on average less to the provision of the public good than *MAOA-H* subjects if variants of *MAOA* had an effect on cooperative behavior. Second, carriers of the low activity alleles would hold rather negative beliefs about the cooperativeness of their peers, thus displaying a pessimistic attitude. Third, with respect to the gene-environment interaction, *MAOA-L* should have a stronger impact on contribution decisions in earlier stages than in later stages. Thus, we would expect that in the initial stage, the gene should play a dominant role in explaining contributions as well as beliefs, whereas the genetic influence should diminish over time.

Experiments including real incentives have the advantage of measuring individual innate characteristics in a population under highly controlled conditions. Thereby, we assessed whether *MAOA* has some predictive power for contribution decisions as well as belief formation over the cooperativeness of others. Furthermore, we studied a gene-environment interaction in the context of an economic experiment, which has previously only been done by McDermott *et al.* [34]. We investigated an environment in which the amount of information about others' behavior increases over time. Thus, we added a dynamic perspective to the discussion by assessing the impact of the gene on contributions and beliefs dependent on various levels of information about the behavior of others and updated beliefs about others' behavior. As far as we know, this is the first study to explore a main gene effect in a public goods experiment and provides new insights into cooperation preferences.

## Results

In the first round, participants contributed approximately half of their endowment (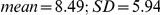). Contributions as well as beliefs declined constantly over the 10 rounds. On average, the actual contributions were slightly lower than the beliefs about how much the others would contribute (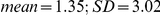) and the difference was significantly different from 0 (one-sample t-test, 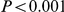). Furthermore, contributions correlated positively with beliefs (Spearman rank correlation test, 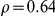, 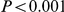). The two latter facts indicate that most of the participants were *imperfect conditional cooperators* with a slight self-serving bias. In other words, they contributed slightly less than what they thought the others would. Overall, the data presented here replicated the results commonly obtained in public goods experiments. Furthermore, we could verify our assumption that the first stage is different from subsequent ones in that players had to rely on their innate beliefs about others' contributions. Following Fischbacher and Gächter [21], we investigated the dynamics of the belief formation process using an econometric model. In particular, we analyzed the role of the initial belief across stages (Table S1). As hypothesized, the initial belief had a significant influence in the second stage, while the coefficient decreased towards zero and became insignificant in stage three and four.

### Behavior of male subjects

**Figure 1:**
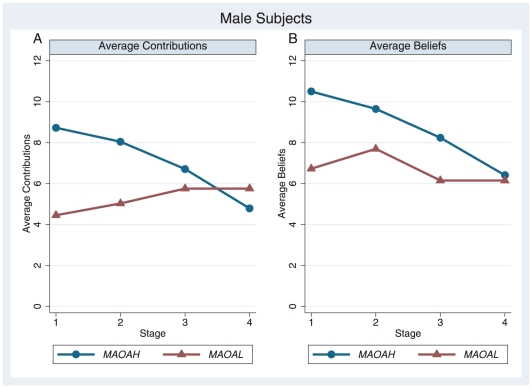
Dynamics of contributions and beliefs of male subjects. A: Average contributions over four stages. B: Average beliefs over four stages.

Both *MAOA-H* and *MAOA-L* subjects were on average conditional cooperators: both groups exhibited similarly high and significant correlations between contributions and beliefs (correlation coefficient: *MAOA-H* 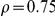, 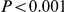; *MAOA-L* 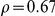, 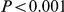). Furthermore, no significant difference regarding the deviation of the contributions from the beliefs was found between *MAOA-H* and *MAOA-L* carriers (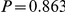). Thus, subjects with low activity *MAOA* alleles were on average imperfect conditional cooperators, too; we did not observe a genetic susceptibility for violation of the prevalent social norm. Yet, as will subsequently be shown, an impact of allelic variation observed when mapping the contributions and beliefs over different levels of environmental variation. In order to identify gene-environment interactions, participants' contributions and beliefs were subjected to a 2 (*MAOA-H* versus *MAOA-L*)×4 (stages) ANOVA, with repeated measures on the latter factor. The results regarding actual contribution (Figure 1A) showed no significant main effect for allelic variation (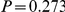) but a significant interaction effect between allelic variation and contributions over the stages (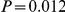). This indicates that the differences in the contributions between high and low carriers of *MAOA* were dependent on the level of information about the others' contributions. Equivalent effects were obtained when all 10 periods were examined (Text S3). Significant interaction effects for within-subjects contrasts (difference) were found in the final stage compared to the previous stages (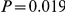), but not for the remaining stages (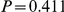; 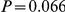). Planned follow-up independent t-tests (Table 1) showed that *MAOA-L* carriers contributed significantly less in stage one (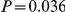) and two (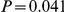,) but not in stage three (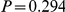) and four (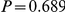). Non-parametric test results were equivalent (Text S4). Overall, the results provided first evidence for a gene-environment interaction regarding the participants' contributions: in an environment with a low amount of information about others' contributions (stage 1 and 2), *MAOA-L* carriers contributed significantly less to the public good; however, in later stages, contributions were independent of allelic variation. With regard to the beliefs about others' contributions (Figure 1B), a significant main effect for allelic variation (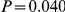) as well as a significant interaction effect between allelic variation and contributions over the stages (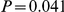) was found. Using all 10 periods replicates the results at the 10%-level (Text S3). Significant interaction effects for within-subjects contrasts (difference) were found in the final stage compared to the previous stages (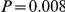), but not for the remaining stages (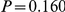; 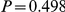). The follow-up independent t-tests (Table 1) revealed that *MAOA-L* carriers exhibited significantly lower beliefs about others' contribution in stage one (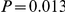), stage two (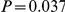), and stage three (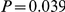), but not in stage four (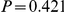). Non-parametric test results were equivalent (Text S4). The results regarding the beliefs about the counterparts' contributions largely resembled the results found for the actual contributions. It seems, however, that expectations about others' contributions were more susceptible to allelic variations as reflected in the significant main effect. Furthermore, the beliefs also appeared to be more insensitive to environmental variations as convergent beliefs between *MAOA-L* and *MAOA-H* carriers were only observed in the final stage.

**Table 1:** Average contributions and beliefs of male subjects.

	average contribution			average belief		
	*MAOA-L*	*MAOA-H*	*MAOA-H* - *MAOA-L*	*MAOA-L*	*MAOA-H*	*MAOA-H* - *MAOA-L*
	mean (SD)	mean (SD)	Difference	mean (SD)	mean (SD)	Difference
stage 1	4.45 (6.56)	8.73 (5.99)	4.27 ( 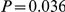 )	6.73 (4.54)	10.50 (2.87)	3.77 ( 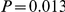 )
stage 2	5.03 (4.60)	8.05 (4.53)	3.02 ( 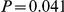 )	7.70 (2.94)	9.65 (2.82)	1.95 ( 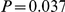 )
stage 3	5.76 (4.50)	6.71 (4.80)	0.95 ( 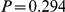 )	6.15 (2.97)	8.24 (3.15)	2.09 ( 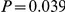 )
stage 4	5.76 (5.24)	4.79 (5.25)	−0.97 ( 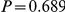 )	6.15 (4.48)	6.41 (2.82)	0.258 ( 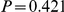 )
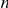	11	22		11	22	

*Notes:* P-values refer to one-sided T-test results.

### Behavior of female subjects

**Figure 2:**
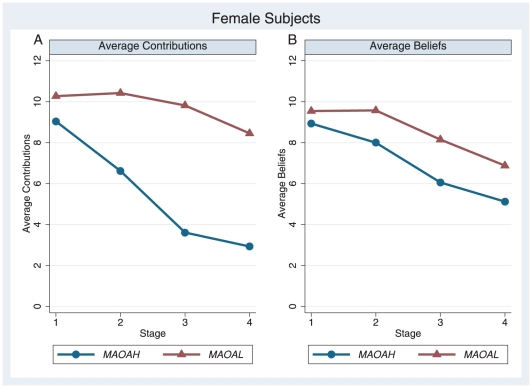
Dynamics of contributions and beliefs of female subjects. A: Average contributions over four stages. B: Average beliefs over four stages.

In line with previous studies, 3/4- and 4/4-genotype females were assigned to the high activity group and 3/3 genotype females to the low activity group. This classification was also supported empirically (Text S5 and Figure S1). Both low and high activity carriers exhibit a significant positive correlation between beliefs and actual contributions (correlation coefficient: *MAOA-H* 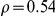, 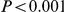; *MAOA-L* 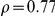, 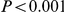), indicating a tendency for imperfect conditional cooperation. Yet, female *MAOA-L* carriers on average show a significant higher tendency for other-serving behavior (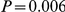). With regard to contributions, a significant main effect for allelic variation (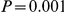) as well as a significant interaction effect between allelic variation and contribution over the stages (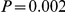) was found (Figure 2A); equivalent results were obtained when using 10 periods (Text S3). Interaction effects for within-subject contrasts (difference) were significant from stage two to stage one (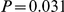) and from stage three to the previous stage (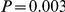); however, not from stage four to the previous stage (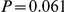). Analogous to males, differences in the contributions between high and low carriers of *MAOA* were mediated by the amount of information about others' cooperativeness, providing further evidence for a gene-environment interaction. In contrast to the male group, however, the female *MAOA-L* carriers contributed significantly more to the public good. A further difference between female and male participants was revealed by the follow-up independent t-tests (Table 2). While no significant difference in the contribution to the public good was found in stage one (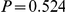) for female *MAOA-L* carriers, females contributed significantly more in stage two (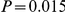), stage three (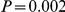), and stage four (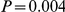). Contrary to men, females exhibiting low activity *MAOA* contributed not only more, but also reacted to changes in environmental conditions in an opposite way.

**Table 2:** Average contributions and beliefs of female subjects.

	average contribution			average belief		
	*MAOA-L*	*MAOA-H*	*MAOA-H* - *MAOA-L*	*MAOA-L*	*MAOA-H*	*MAOA-H* - *MAOA-L*
	mean (SD)	mean (SD)	Difference	mean (SD)	mean (SD)	Difference
stage 1	10.27 (5.00)	9.04 (5.88)	1.23 ( 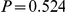 )	9.55 (3.01)	8.94 (3.61)	0.06 ( 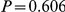 )
stage 2	10.42 (4.70)	6.61 (4.49)	3.81 ( 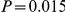 )	9.58 (2.66)	8.00 (2.42)	1.58 ( 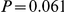 )
stage 3	9.82 (5.13)	3.61 (2.92)	6.21 ( 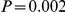 )	8.15 (2.59)	6.06 (2.12)	2.09 ( 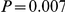 )
stage 4	8.45 (4.92)	2.92 (2.70)	5.53 ( 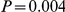 )	6.88 (2.37)	5.12 (1.81)	1.76 ( 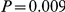 )
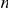	11	47		11	47	

*Notes:* P-values refer to two-sided T-test results.

With regard to the beliefs about others' contributions (Figure 2B), no significant interaction effect was observed (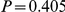). Likewise, the interaction effects for within-subject contrasts (difference) were non-significant (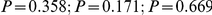). Yet the female *MAOA-L* carriers hold significantly higher beliefs regarding the contribution of others as reflected in the main effect for allelic variation (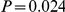). The follow-up independent t-tests (Table 2) show that the difference is driven by the final stages. Similarly to the contributions, significant differences were found in the final two stages (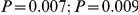) but not in the first two stages (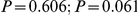). Additionally, *MAOA-L* female carriers contributed on average even higher amounts than the amount they expected others to provide. Although the difference is not statistically significant (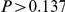, t-test, two-sided), this finding provides a first hint for a potential correlation between MAOA-L and altruistic behavior among females.

Overall, the results for female participants substantiated the influence of allelic variation on contribution behavior and beliefs as well as the interaction between allelic variation and environmental condition. The female participants, however, exhibited opposite patterns compared to males. As for male participants, our primary group of interest, the effect of allelic variation was more dominant in environmental conditions of low information about others' behavior; the opposite seemed to hold true for female participants. Furthermore, relative to male *MAOA-L* carriers, the female participants with a low activity level of MAOA contributed more to the public good and also held higher beliefs about others' contributions.

## Discussion

By focusing on a particular candidate gene and its association with individual behavior in experimental games, we identified causal pathways through which genetic variations influence economic decision-making. We investigated the genetic basis of cooperative behavior in a public goods experiment. It has previously been suggested that a neural network is involved in social norm compliance [39], [40] and that genetic mechanisms regulating dopaminergic and serotonergic synaptic transmission might contribute to the explanation of social behavior. Among the genes related to neurotransmitters, the *MAOA* gene is a prime candidate as it encodes an enzyme that degrades neurotransmitters with the number of tandem repeats polymorphism impacting transcriptional efficiency. Given a large body of research suggesting that *MAOA-L* is associated with antisocial behavior (leading to its nickname “warrior gene”), we hypothesized that male carriers of the low efficiency allele would rather free-ride than cooperate, would contribute smaller shares of their endowments (if any), and would be more susceptible to violations of the social norm of conditional cooperation. Furthermore, we hypothesized that *MAOA-L* males would hold more pessimistic expectations about the cooperativeness of others.

Our results support the view that a genetic source of individual variation in human cooperation exists: *MAOA-L* male carriers contributed significantly less to the public good than *MAOA-H* subjects in the first and early time periods. With additional information about the others' behavior, the role of *MAOA* diminished. Although both genotypes are on average conditional cooperators, they had significantly different expectations in the first rounds: *MAOA-L* subjects were on average conditional cooperators with pessimistic beliefs about others' contributions, *MAOA-H* individuals were rather characterized as optimists. Thus, rather than consciously violating the prevalent social norm of conditional cooperation, carriers of the low efficient alleles remarkably held lower initial expectations about their social environment and this pessimistic view might have driven different behavior between genotypes. Although not easy to determine, twin and adoption studies showed that pessimism itself is a heritable trait, with heritability estimates of about 25% [41]. Whether *MAOA*, or other genes, directly affect pessimistic or optimistic attitudes in general has yet to be determined.

Although there is still no consensus on the explanation of the well-known decay in contributions in repeated-round settings, the idea that the clue is to be sought in conditional cooperative behavior has received particular attention [1], [20], [21], [42]. It has long been argued [42] that the majority of individuals in public goods games uses information about the average group contributions as an anchor for its own future contribution: those who are above (below) the average in one round decrease (increase) their contribution in the following round. Our results are in accordance with Neugebauer *et al.* [35] who evaluated several competing hypotheses and found that the one of conditional cooperation and adaptive belief learning is the only viable one. In a recent study [21], it has been shown that it is the “imperfectness” of conditional cooperation that leads to the decay: people do not perfectly match others' contributions, but contribute a little less than what they observe or expect. This “self-serving bias” leads to a fall in contributions over time. Indeed, if an entire group consists of imperfect conditional cooperators, group contributions will decline over time. The presence of free-riders is not necessary for this scenario, although it would speed up the downward trend.

Given a scenario of conditional cooperators (be it imperfect or perfect ones) who differ in their initial beliefs, Chaudhuri [43] suggests two types of players: those with optimistic and those with pessimistic initial beliefs. Whereas optimists start out with high contributions, those with pessimistic beliefs contribute less in the first rounds. Over time, both types are able to observe the contributions of their peers and adapt their own contributions accordingly: pessimists increase their contributions, while optimists decrease them. Additionally, we found a genetic basis for the differences in the distribution of initial beliefs: *MAOA-L* carriers are the pessimists and *MAOA-H* carriers hold more optimistic beliefs. As *MAOA-L* genotypes on average only account for one third in a given population and their contribution increases are too small to offset the decrease of optimistic *MAOA-H* carriers, we can observe the stylized fact of a continuous decay over time.

Up to now, little is known about the origins of belief heterogeneity. This is the first work which studies the association between a particular gene and beliefs about the cooperativeness of peers. Our research suggests that there are robust exogenous sources of variation in initial beliefs that converge over time and may so explain converging contributions to the public good. We are not aware of any study which has analyzed a gene-environment-interaction like the one studied here: we investigated not only static behavior, but the dynamics of adapting contributions and beliefs in the presence of increasing information about others' behavior. Our research has been driven by the hypothesis that a gene alone does not determine complex social behavior. Instead, we tried to contribute to the understanding of the interplay between gene effects, environmental effects, and gene-environment-interaction effects in causing variation in economic phenotypes.

Our study revealed considerable gender differences with respect to genetically modulated voluntary provision of public goods. Whereas males acted essentially as hypothesized in our experiment, with *MAOA-L* carriers contributing less to the provision of the public good compared to *MAOA-H* carriers, females behaved contrariwise. However, recent studies on gender differences in cooperation and competition demonstrated that in their social environment females on average cooperate more than men [44]. On the other hand, the genetic influence is more pronounced in women in later rounds. These behavioral patterns may be due to evolutionary selected different strategies of the sexes to enhance their reproductive fitness, demanded by historically different social environments of both males and females. The social psychology of women is characterized by higher genetic relatedness and different kinds of need such as defending their offspring and creating a supportive social network [45]. Genes in general may have opposing behavioral associations in men and women. On the one hand, sex hormones such as testosterone and estrogen and their receptors act gender-specific on gene regulation and subsequent development of the brain [46], [47]. For example, high testosterone exposure in early life leads to more male-typical behavior and brain structure, which leads to differing responses of the sexes to environmental stimuli later in life [47]. For instance, empathy, a personality characteristic which is on average higher in females than in males, seems to be reduced by higher testosterone levels [48]. Another possible explanation for the different behavior of males and females in our study might be due to the influence of estrogens on the differentiation of dopaminergic neurons in the brain and its role as a neuromodulative reagent [49]. On the other hand, X-chromosomal genes such as MAOA have specific effects on social cognition and emotional regulation [50]. Males are prone to be influenced by X-linked haploidy and to show deficits in mental abilities because of the direct impact of genetic variations carried in the haploid state [50]. Particularly, *MAOA* has been shown to have differing associations. *MAOA* association studies consistently show a sex-by genotype interaction [13], [27], [51], although the evidence where it stemmed from is ambiguous: one group of studies reported that behavioral effects of *MAOA* variation were more pronounced in males than in females in both animal [52] and human [8], [27] studies. Another group of studies, however, described opposing patterns of association in men and women [53], [54]. For example, women carrying the *MAOA-H* showed a higher risk of being high alcohol consumers, whereas among men, *MAOA-L* was related to higher alcohol consumption [51]. Among girls with psychosocial risk, *MAOA-L* conferred an increased risk for criminal behavior, whereas among men, the short (3-repeat) allele and social risk interacts to predict criminal activity [55]. Meta-analyses showed highly inconsistent gender differences in social preferences [36], suggesting that women might be more sensitive to the context of social decision-making experiments.

Rather than using self-reported data, we studied the actual behavior of participants in a controlled laboratory experiment, thus complementing mounting survey results about the role of *MAOA*. In doing so, we made a contribution to an emerging literature using economic experiments to examine the role genetic variation plays in explaining behavioral heterogeneity [56]–[61]. Moreover, we contributed to the emerging literature on the neuroeconomics of decision making in general [62], [63]and to a new research area called genoeconomics [23] in particular. The purpose of genoeconomics is to investigate how individual genetic variation interacts with the social environment to influence economic traits. Indeed, different alleles of *MAOA* seem to influence individual heterogeneity in cooperativeness and expectations thereof depending on the dynamics of others' behavior. Our results suggest that social scientists might take seriously the idea that genes too contribute to variability in cooperation.

## Materials and Methods

### Ethics Statement

Informed written consent was obtained from all subjects for participation in the economic experiment and collection of buccal cells. The study was approved by the ethics committee of the University of Trier.

### Experimental Design and Procedures

A total of 96 students (60 women and 36 men) who were recruited using ORSEE [64] participated voluntarily in this study. The subjects (mean age 24.8+/−3.3) were all students from different disciplines at the University of Trier. We conducted four sessions with 24 subjects each in June and July 2010. A post-experimental questionnaire confirmed that participants were largely unacquainted with one another. Following a standard rule in economic experiments, subjects were paid according to their decisions and those by group members. The repeated public goods game was part of a sequence of tasks. Before subjects played it they had to fill in a contribution table therewith playing a strategic public goods game. Fischbacher and Gächter [21] found that the experimental sequence does not have any behavioral effect in these games. Since we used exactly their experimental protocol, we refer the reader to the original source and to the Appendix for any details. The repeated public goods game lasted about 45 minutes and subjects earned on average 12.32 Euro (roughly $18, including a show-up fee of 5 Euro).

The computerized experiments were conducted in the computer lab of the University of Trier using the software z-Tree [65]. Subjects were randomly allocated a computer terminal on a local network that was monitored and controlled from the experimenter's terminal. To ensure that all activity in the experiment was completely anonymous, subjects were separated by a dividing wall, group composition changed randomly every period, and no subject would ever learn about paired group members or their history of play. These procedures eliminated concerns for reputation or revenge. Subjects received written instructions which were read aloud in order to make sure that everybody understood that the instructions were identical for all participants. To make sure that subjects understood the rules of the game and the incentives, they had to answer ten control questions which all had to be answered correctly by all subjects before proceeding. After the experiment, subjects donated buccal cells for genotyping. Experimental instructions are reported in Text S2.

### Statistical Analysis

In line with the directional (undirectional) hypotheses we reported one-tailed (two-tailed) test statistics for the planned follow-up t-tests with regard to the male (female) participants. The data were tested for Gaussian distribution (Kolmogorov-Smirnov test) and sphericity (Mauchly-W). As departures from normality were observed in some instances, we additionally reported the results of the non-parametric tests in the appendix. When sphericity was not assumed (Mauchly-W
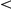
0.05), the Greenhouse-Geisser correction was applied. Finally, the ten rounds of the experiment were aggregated to four stages in order to improve statistical properties of the data and clarity of the results. The first round was defined as stage one as the participants had no information about others' contribution. The remaining nine rounds were equally divided into three stages and aggregated by taking the mean of three rounds. The results for the non-aggregated ten rounds are, however, reported in the appendix.

## Supporting Information

Figure S1 **Dynamics of contributions and beliefs of female subjects separated by genotype.** A: Average contributions over four stages for 3/3, 3/4, and 4/4 genotypes. B: Average beliefs over four stages for 3/3, 3/4, and 4/4 genotypes.
(EPS)

Text S1 **Genotyping.**
(PDF)

Text S2 **Experimental Instructions.**
(PDF)

Text S3 **Results from Ten Rounds.**
(PDF)

Text S4 **Non-parametric Tests.**
(PDF)

Text S5 **Female Genotype Groups.**
(PDF)

Table S1 **Belief Formation Process.**
(PDF)
