# Supplementary material for: Genetic Susceptibility for Individual Cooperation Preferences: The Role of Monoamine Oxidase A Gene (MAOA) in the Voluntary Provision of Public Goods
Source: PLoS One. 2011 Jun 16;6(6):e20959. doi: 10.1371/journal.pone.0020959 (PMC3116851; doi:10.1371/journal.pone.0020959)
Supplement: Text S1 — Genotyping. (PDF) [file pone.0020959.s002.pdf]

Supplementary Information Text S1

Genotyping

DNA was obtained from buccal cells followed by a mouthwash with Listerine (Qiagen Gentra Puregene Buccal Cell Kit, Hilden, Germany). PCR was performed in 50 μl reactions with a total DNA concentration of 100 ng, 1.5 mM MgCl2, 10 pmol of each primer, 0.2 mM dNTPs and 1.25 U Hot Star *Taq* Polymerase (Qiagen). *MAOA* primer sequences were previously described as [1]: *MAOA* Fwd (5'-ACAGCCTGACCGTGGA-GAAG-3') and *MAOA* Rev (5'GAACGGACGCTCCATTCGGA- 3'). Thermal cycling was carried out using the following conditions: pre-step 15 min at 95°C, 5 min denaturing step at 94°C, 40 cycles at 94°C for 30 sec, 63°C for 40 sec, 72°C for 30 sec and a final extension phase at 72°C for 7 min. PCR products were separated on a 2 % agarose gel and visualised with ethidium bromide under UV light. The PCR products resulted in fragments of 291 bp, 321 bp, 336 bp, 351 bp and 381 bp, corresponding to the 2-, 3-, 3.5-, 4- and 5-repeat alleles, respectively. Group definition for *MAOA-L* (low activity) and *MAOA-H* (high activity) were previously described [2].

References

[1] Sabol SZ, Hu S, Hamer D (1998) A functional polymorphism in the monoamine oxidase A gene promoter. Hum Genet 103: 273-279.

[2] Reif A, Scarpini E, Venturelli E, Töpner T, Fenoglio C, et al. (2008) The functional MAOA-uVNTR promoter polymorphism in patients with frontotemporal dementia. Eur J Neurol 15: 637-639.
