# Supplementary material for: Genetic Susceptibility for Individual Cooperation Preferences: The Role of Monoamine Oxidase A Gene (MAOA) in the Voluntary Provision of Public Goods
Source: PLoS One. 2011 Jun 16;6(6):e20959. doi: 10.1371/journal.pone.0020959 (PMC3116851; doi:10.1371/journal.pone.0020959)
Supplement: Text S2 — Experimental Instructions. (PDF) [file pone.0020959.s003.pdf]

Supplementary Information Table S2

Experimental Instructions

[As we have replicated the experiment by Fischbacher and Gächter [1], these instructions follow the original ones. This is a translation of the German written instructions which were used in the experiment. The original one is available upon request.]

Instructions You are now taking part in an economics experiment financed by a research grant from the University of Trier. If you read the following instructions carefully, you can, depending on your decisions, earn some more money in addition to the 5 Euro, which you can keep in any case. The entire amount of money, which you earned with your decisions will be added up and paid to you in cash at the end of the experiment. These instructions are solely for your private information. You are not allowed to communicate during the experiment. If you have any questions, please ask us. Violation of this rule will lead to the exclusion from the experiment and all payments. If you have questions, please raise your hand. A member of the experimenter team will come to you and answer them in private. We will not speak of Euro during the experiment, but rather of points. Your whole income will first be calculated in points. At the end of the experiment, the total amount of points you earned will be converted to Euro. All participants will be divided in groups of four members. Except for us, the experimenters, no one knows who is in which group. We describe the exact experiment process below.

The decision situation You will learn how the experiment will be conducted later. We first introduce you to the basic decision situation. You will find control questions at the end of the description of the decision situation that help you to understand the decision situation. You will be a member of a group consisting of 4 people. Each group member has to decide on the allocation of 20 points. You can put these 20 points into your private account or you can invest them fully or partially into a project. Each point you do not invest into the project, will automatically remain in your private account.

Your income from the private account You will earn one point for each point you put into your private account. For example, if you put 20 points into your private account (and therefore do not invest into the project) your income will amount to exactly 20 points out of your private account. If you put 6 points into your private account, your income from this account will be 6 points. No one, except you, earns something from your private account.

Your income from the project Each group member will profit equally from the amount you invest into the project. On the other hand, you will also get a payoff from the other group members' investments. The income for each group member will be determined as follows: *Income from the project = sum of all contributions* x 0.4. If, for example, the sum of all contributions to the project is 60 points, you and the other members of your group each earn 60 x 0.4 = 24 points out of the project. If four members of the group contribute a total of 10 points to the project, you and the other members of your group each earn 10 x 0.4 = 4 points.

Total income Your total income is the sum of your income from your private account and that from the project: *Income from your private account (=20 - contribution to the project) + Income from the project (= 0.4* x *sum of all contributions to the project) = Total income.*

Control questions Please answer the following control questions. They will help you to gain an understanding of the calculation of your income, which varies with your decision about how you distribute your 20 points. Please answer all questions and enter the appropriate number. You are allowed to use the integrated calculator. To do so, double click on the calculator icon in the bottom left corner on your screen.

Instructions for the C-Experiment The experiment lasts 10 periods, in which you and the other group members have to make decisions. As in the other experiment, every group consists of 4 people. The formation of the group changes at random after every period. So your group consists of different people in all 10 periods. The whole experiment is finished after these 10 periods,. The decision situation is the same as that described on page 2 of the instructions of the previous experiment. Each member of

the group has to decide about the usage of the 20 points. You can put these 20 points into your private account or you can invest them fully or partially into a project. Each point you do not invest into the project is automatically placed into your private account. Your income will be determined in the same way as before. Reminder: *Income from your private account (=20 - contribution to the project) + Income from the project (= 0.4* x *sum of all contributions to the project) = Total income.*

During the experiment, we do not speak about Euro, but points. Your total profit will be calculated in points. At the end of the experiment, the points will be converted in Euro at an exchange rate of: 1 point = 3 Cent.

The decision screen, which you will see in every period, looks like this:

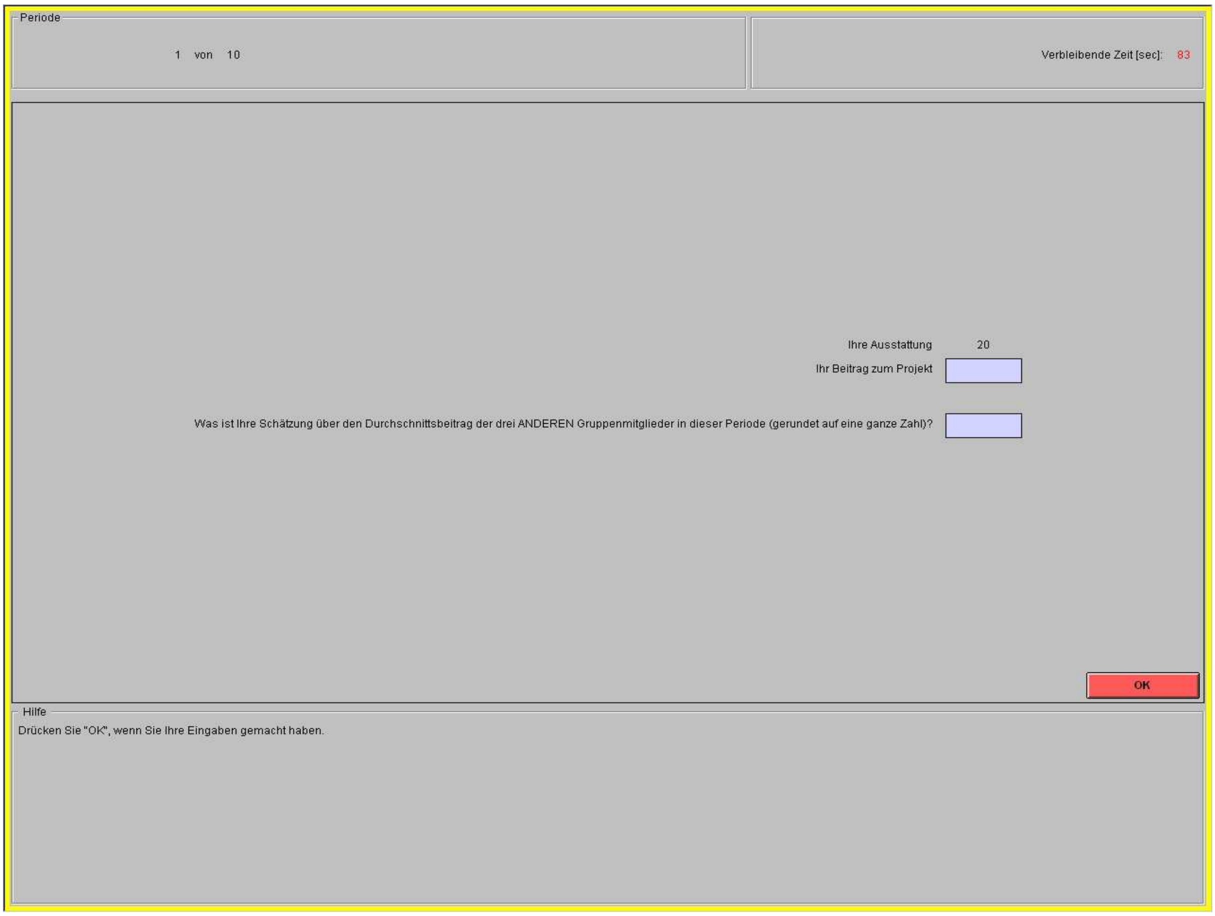

As you can see, you have to make two inputs:

1. First you have to decide on your contribution to the project, that is, you have to decide how many of the 20 points you want to contribute to the project, and how many points you want to put into your private account. This decision is the same as the unconditional contribution of the previous experiment. You only make unconditional decisions in this experiment. There is no contribution table.

2. Afterwards, you have to estimate the average contribution to the project (rounded to an integer) of the other three group members of this period. You will be paid for the accuracy of your estimate:

- If your estimate is exactly right (that is, if your estimate is exactly the same as the actual average contribution of the other group members), you will get 3 points in addition to your other income from the experiment.
- If your estimate deviates by one point from the correct result, you will get 2 additional points.
- A deviation by 2 points still earns you 1 additional point.
- If your estimate deviates by 3 or more points from the correct result, you will not get any additional points.

References

[1] Fischbacher U, Gächter S (2010) Social preferences, beliefs, and the dynamics of free riding in public goods experiments. Am Econ Rev 100: 541-556.
