# Supplementary material for: Genetic Susceptibility for Individual Cooperation Preferences: The Role of Monoamine Oxidase A Gene (MAOA) in the Voluntary Provision of Public Goods
Source: PLoS One. 2011 Jun 16;6(6):e20959. doi: 10.1371/journal.pone.0020959 (PMC3116851; doi:10.1371/journal.pone.0020959)
Supplement: Text S3 — Results from Ten Rounds. (PDF) [file pone.0020959.s004.pdf]

Supplementary Information Table S3

Results from Ten Rounds

The ten rounds of the experiment were aggregated to four stages in order to improve statistical properties of the data and clarity of the results. Employing ten stages, however, leads to identical results with regard to main and interaction effects. For the male participants' contributions, a significant interaction effect ($P = 0.042$), but no significant main effect for allelic variation ($P = 0.403$) was found. With regard to beliefs, the main ($P = 0.092$) and the interaction effect ($P = 0.090$) are significant at the 10%-level. Likewise, the results of the female participants from the ten rounds also resembled the results for the four stages. With regard to contributions, both the main and the interaction effect were significant ($P < 0.001; P = 0.007$). The beliefs about the contribution of the others showed a significant main effect ($P = 0.009$) and no significant interaction effect ($P = 0.684$).
