# Supplementary material for: Genetic Susceptibility for Individual Cooperation Preferences: The Role of Monoamine Oxidase A Gene (MAOA) in the Voluntary Provision of Public Goods
Source: PLoS One. 2011 Jun 16;6(6):e20959. doi: 10.1371/journal.pone.0020959 (PMC3116851; doi:10.1371/journal.pone.0020959)
Supplement: Text S4 — Non-parametric Tests. (PDF) [file pone.0020959.s005.pdf]

Supplementary Information Table S4

Non-parametric Tests

Due to departures from normality in some instances and a small sample size, we additionally calculated the Wilcoxon rank-sum test. The results resembled the results obtained from t-tests. For male participants' contributions, we again found significant differences in the first two stages ($Z = -1.91, P = 0.029; Z = -1.76, P = 0.041$), but not in the final two ($Z = -0.42, P = 0.346; Z = 0.33, P = 0.625$). The differences in beliefs about the others' contributions were significant in stage one ($Z = -2.48, P = 0.006$), stage two ($Z = -1.97, P = 0.024$), stage three ($Z = -1.78, P = 0.038$), and not in stage four ($Z = -0.86, P = 0.198$). For female participants, the results obtained from the Wilcoxon rank-sum test replicated the t-tests. The difference in contributions were significant in stage two ($Z = -2.25, P = 0.024$), three ($Z = -3.90, P < 0.001$), and four ($Z = -3.65, P < 0.000$), but not in stage one ($Z = -0.49, P = 0.618$). The differences in beliefs, which also correspond to the t-test, were significant in the final two stages ($Z = -2.76, P = 0.006; Z = -2.34, P = 0.19$) and not in the first two stages ($Z = -0.14, P = 0.889; Z = -1.810, P = 0.070$).
