# Supplementary material for: Genetic Susceptibility for Individual Cooperation Preferences: The Role of Monoamine Oxidase A Gene (MAOA) in the Voluntary Provision of Public Goods
Source: PLoS One. 2011 Jun 16;6(6):e20959. doi: 10.1371/journal.pone.0020959 (PMC3116851; doi:10.1371/journal.pone.0020959)
Supplement: Text S5 — Female Genotype Groups. (PDF) [file pone.0020959.s006.pdf]

Supplementary Information Text S5

Female Genotype Groups

Most, but not all, studies assign the 3/4- and 4/4-genotype females to the high activity group and the 3/3-genotype females to the low activity group. Pooling the 3/4- and 4/4-genotype was also supported by our data. Multiple comparisons (Scheffe procedure) showed that if any significant differences in contributions or beliefs between the three groups exist, they were found between 3/3 and 3/4 and/or 3/3 and 4/4 but, never between 3/4 and 4/4. For contributions we found: significant difference at the 10%-level between 4/4 and 3/3 ($P = 0.054$) in stage two; significant differences between 3/4 and 3/3 ($P < 0.001$) as well as 4/4 and 3/3 ($P < 0.001$) in stage three; significant differences between 3/4 and 3/3 ($P < 0.001$) as well as 4/4 and 3/3 ($P < 0.001$) in stage four. For beliefs we found: a significant difference between 4/4 and 3/3 ($P = 0.036$) and a significant difference at the 10 % level between 3/4 and 3/3 ($P = 0.058$) in stage three; significant difference between 4/4 and 3/3 ($P = 0.041$) and a significant difference at the 10% level between 3/4 and 3/3 ($P = 0.076$) in stage four. The 3/4-genotype takes on a middle position and differences were more pronounced between 3/3 and 4/4; however, the results support the necessity to assign 3/4- and 4/4-genotype females to the high activity group, as done in previous studies. Complete results of the multiple comparisons can be obtained from the authors upon request.
