# Supplementary material for: Genetic Susceptibility for Individual Cooperation Preferences: The Role of Monoamine Oxidase A Gene (MAOA) in the Voluntary Provision of Public Goods
Source: PLoS One. 2011 Jun 16;6(6):e20959. doi: 10.1371/journal.pone.0020959 (PMC3116851; doi:10.1371/journal.pone.0020959)
Supplement: Table S1 — Belief Formation Process. (PDF) [file pone.0020959.s007.pdf]

Supplementary Information Table S1

Belief formation process

	Dependent variable: Belief		
Stage	2	3	4
Contribution Others (t-1)	0.424	0.438	0.355
	(0.040)***	(0.098)**	(0.066)**
Belief (t-1)	0.376	0.433	0.661
	(0.045)***	(0.044)***	(0.036)***
Belief (t=1)	0.114	0.082	0.059
	(0.025)**	(0.043)	(0.042)
Constant	0.783	0.338	-0.348
	(0.237)**	(0.332)	(0.565)
R^2	0.52	0.55	0.54
Observations	288	288	288

OLS estimates with robust standard errors (clustered by session) in parantheses.

* significant at 10%; ** significant at 5%; *** significant at 1%
